# Immune Escape in Glioblastoma Multiforme and the Adaptation of Immunotherapies for Treatment

**DOI:** 10.3389/fimmu.2020.582106

**Published:** 2020-10-15

**Authors:** Joshua R. D. Pearson, Stefania Cuzzubbo, Simon McArthur, Lindy G. Durrant, Jason Adhikaree, Chris J. Tinsley, A. Graham Pockley, Stephanie E. B. McArdle

**Affiliations:** ^1^The John van Geest Cancer Research Centre, School of Science and Technology, Nottingham Trent University, Nottingham, United Kingdom; ^2^Centre for Health, Ageing and Understanding Disease (CHAUD), School of Science and Technology, Nottingham Trent University, Nottingham, United Kingdom; ^3^Université de Paris, PARCC, INSERM U970, Paris, France; ^4^Laboratoire de Recherches Biochirurgicales (Fondation Carpentier), Assistance Publique-Hôpitaux de Paris (AP-HP), Hôpital Européen Georges Pompidou, Paris, France; ^5^Institute of Dentistry, Barts & the London School of Medicine & Dentistry, Blizard Institute, Queen Mary, University of London, London, United Kingdom; ^6^Scancell Ltd, Biodiscovery Institute, University of Nottingham, Nottingham, United Kingdom; ^7^Academic Oncology, Nottingham University NHS Trusts, City Hospital Campus, Nottingham, United Kingdom

**Keywords:** GBM - Glioblastoma multiforme, immune escape, immunotherapy, combinatorial therapy, treatment, overview

## Abstract

Glioblastoma multiforme (GBM) is the most frequently occurring primary brain tumor and has a very poor prognosis, with only around 5% of patients surviving for a period of 5 years or more after diagnosis. Despite aggressive multimodal therapy, consisting mostly of a combination of surgery, radiotherapy, and temozolomide chemotherapy, tumors nearly always recur close to the site of resection. For the past 15 years, very little progress has been made with regards to improving patient survival. Although immunotherapy represents an attractive therapy modality due to the promising pre-clinical results observed, many of these potential immunotherapeutic approaches fail during clinical trials, and to date no immunotherapeutic treatments for GBM have been approved. As for many other difficult to treat cancers, GBM combines a lack of immunogenicity with few mutations and a highly immunosuppressive tumor microenvironment (TME). Unfortunately, both tumor and immune cells have been shown to contribute towards this immunosuppressive phenotype. In addition, current therapeutics also exacerbate this immunosuppression which might explain the failure of immunotherapy-based clinical trials in the GBM setting. Understanding how these mechanisms interact with one another, as well as how one can increase the anti-tumor immune response by addressing local immunosuppression will lead to better clinical results for immune-based therapeutics. Improving therapeutic delivery across the blood brain barrier also presents a challenge for immunotherapy and future therapies will need to consider this. This review highlights the immunosuppressive mechanisms employed by GBM cancers and examines potential immunotherapeutic treatments that can overcome these significant immunosuppressive hurdles.

## Introduction

Glioblastoma multiforme (GBM, WHO grade 4) is the most frequently occurring primary brain tumor. Although primarily a disease associated with old age, it can also occur in children. The prognosis for GBM patients is poor and the disease is almost uniformly fatal with only around 5% of patients surviving for a period of 5 years after diagnosis (1). The current course of therapy for GBM patients is surgical resection of the tumor (where possible) followed by concomitant radiotherapy and temozolomide chemotherapy, followed by adjuvant temozolomide. Despite aggressive multimodal therapy, GBM tumors nearly always recur, the majority close to the site of resection (2–4). This recurrence is most likely, and most often, due to the infiltrative nature of GBM making complete resection of tumor cells incredibly difficult. Although progress to improve the surgical removal of tumor cells has been made, such as the use of 5-aminolevulinic acid (5-ALA) which is approved for intraoperative imaging of GBM cells increasing their removal, it is not possible to visualize all individual cancer cells that have migrated further into healthy areas of the brain (5).

GBM tumors are histopathologically characterized by an abundance of poorly differentiated and pleomorphic astrocytes with nuclear atypia and high mitotic activity. GBM tumors are highly vascular and necrosis is often evident within these tumors (6). Metastasis is rarely seen in GBM tumors; however, they are highly invasive, and these tumors employ a plethora of mechanisms to avoid immune detection.

## The Brain as a Unique Immune Environment

In order to understand the complexity of the brain’s interaction with the immune system, the presence of the blood-brain barrier (BBB) needs to be considered and understood. The endothelial cells of the brain vasculature are connected by tight junctions that control the permeability of the endothelium. Although these tight junctions under normal physiological conditions are highly regulated, under inflammatory conditions (such as those in GBM) these junctions are not as tightly connected making the endothelium ‘leaky’ (7). The BBB, a multi-component structure found in the wall of cerebral blood vessels, selectively restricts passage of cells and molecules into the brain from the circulation. The major, but not sole, players in this defense are the endothelial cells of the cerebral vessels, which differ from their peripheral counterparts by the presence of intercellular tight junctions that essentially prevent paracellular transfer of all, but the smallest gases and ions, and the absence of fenestrations and pinocytic mechanisms that restricts bulk transcytosis (8, 9). These features are then reinforced by the presence of numerous efflux transporters that remove xenobiotics and metabolic waste from the brain into the circulation. Beyond the endothelium, the BBB is further composed of a bi-layered basement membrane within which reside pericytes and perivascular macrophages that regulate endothelial function and pose a further barrier to cellular entry, ultimately surrounded by a tight glia limitans formed of astrocyte end-feet that appose and encircle the blood vessel (8, 9).

GBM tumors contain areas of highly metabolic cells that drive local hypoxia, triggering production of vascular endothelial growth factor and angiogenesis (10). This process involves disruption of inter-endothelial tight junctions to permit vascular growth, hence the core of the tumor is associated with a weakened blood-tumor barrier (BTB) with an increased permeability (11, 12). Nevertheless, areas of GBM tumors distal from the hypoxic core, which in diffuse tumors can be a significant proportion, remain behind a BTB that is highly reminiscent of the true BBB, and are thus protected from the entry of chemotherapeutic agents, including most therapeutic antibodies (13). However, these difficulties do not mean that the delivery of effective therapeutics for GBM is futile, and a wide variety of approaches to achieve this are under active exploration.

The brain has traditionally been considered as being an immunoprivileged organ due to a variety of factors, however it is now accepted that there is an active interaction between the brain and the immune system (14, 15). Despite this active immune interaction, the brain is immunologically unique in that immune cells do not freely access the brain parenchyma. Although activated immune cells can cross the BBB, only those specific for antigens within the brain remain there. T cells cross the BBB in a capture, crawl, cross manner with integrins and selectin ligands on T cells binding to selectins and integrin ligands on endothelial cells ‘capturing’ them (16). Leukocytes are then activated by chemokine secretion resulting in their slowing and eventual transmigration. Once T cells have transmigrated, they downregulate their integrin expression and upregulate expression of matrix metalloproteinases (MMPs) enabling them to break down matrix components allowing cell penetration of the brain parenchyma (7). Inflammation within the brain has been shown to lead to an upregulation of adhesion molecules on the BBB endothelial cells (16, 17). The endothelial cells of the brain vasculature do not just control the immune response by physically excluding immune cells, these cells have also been shown to contribute to immunosuppression in GBM. FasL expression has been seen on GBM associated vascular endothelial cells, and the FasL expressed on these cells is linked to a reduced T cell infiltrate, most likely due to the FasL induced death of T cells (18). There are very few immune cells normally present within the brain, however the microglia can act as antigen presenting cells (APCs). The brain traditionally has low major histocompatibility complex (MHC) class I and class II expression meaning that antigen expression is reduced when compared to other tissues (19). It is important to note, however, that GBM cells themselves have been shown to express MHC class I and II molecules meaning that these cells present antigens to antigen specific CD8^+^ and CD4^+^ T cells (20).

Not only does the unique physiology of the brain create an unusual immune environment but it is important to note that the tumors themselves create their own microenvironment. Tumor cells can co-opt stromal cells in order to support their growth and survival (21). The brain extracellular matrix is comprised of proteoglycans, glycoproteins and glycosaminoglycans. In the GBM setting, significant increases in heparan sulphate proteoglycans (HSPG) have been seen in the tumor microenvironment (TME) (22). The increase of HSPGs in the TME leads to greater retention of growth factors such as vascular endothelial growth factor (VEGF) and fibroblast growth factor (FGF), thereby supporting tumor nutrition and growth. The increased local concentration of VEGF within the GBM TME results in upregulation of periostin and tenascin C within blood vessels trapping T cells and preventing tumor penetrance (23).

In the case of GBM, as with many cancers T cells are frequently exhausted and dysfunctional and therefore are inadequate at exerting an anti-tumor immune response. Persistent stimulation of T cells by tumor cells results in T cell senescence as indicated by the presence of CD57 on the surface of T cells (24). CD57 positive T cells can secrete cytokines when stimulated by their cognate peptides however they do not proliferate when stimulated (25). Tumor resident senescent T cells have also been shown to down regulate the co-stimulatory molecules CD27 and CD28 contributing to immune dysfunction, causing changes in APC phenotype such as a down regulation of CD80 and CD86 reducing their ability to stimulate T cells further exacerbating the local immune dysfunction (26). When compared to healthy donors GBM patients have a lower number of circulating CD3^+^ T cells in their peripheral blood mononuclear cells (PBMCs) further indicating a disease related immune dysfunction (27). Glioblastoma multiforme is more frequent in the older population with most cases occurring between the ages of 55 and 60 (28). Increased age is linked to T cell dysfunction; with elderly patients having a higher number of senescent T cells and thymic shrinkage being apparent (24, 29). The chronic stimulation of T cells by tumor cells also leads to the exhaustion of these cells, rendering them ineffective at tumor control. This exhaustion leads to an upregulation of immune checkpoint markers such as PD-1, LAG-3, TIGIT, and CD39 on GBM infiltrating CD8^+^ T cells (30). TILs isolated from murine GBM tumors show impaired cytokine production compared to peripheral T cells, with reduced levels of interferon gamma, tumor necrosis factor alpha and interleukin 2 being detected via flow cytometry when cells are stimulated *in vitro* (30). Transformed tumor cells also compete with other cells within the TME for glucose, GBM cells have an increased rate of glucose uptake when compared to non-transformed cells. T cells within the TME require glucose in order to perform effector functions and therefore the depletion of glucose by tumor cells results in impaired T cell function and exhaustion (31).

## Standard of Care and Immunosuppression

The current standard of care for GBM is maximal surgical resection (where possible) followed by concomitant radiotherapy and temozolomide chemotherapy (32). Patients are also given anti-inflammatory steroids such as dexamethasone to help control peritumoral edema (33). The US Food and Drug Administration (FDA) has also approved the use of tumor treating fields (TTFs) to treat GBMs. This involves using alternating electric fields administered via scalp electrodes to disrupt GBM tumor cell division (34).

Dexamethasone has been shown to lead to the upregulation of the immunosuppressive checkpoint cytotoxic T-lymphocyte-associated protein 4 (CTLA-4) on the surface of T cells, thereby reducing their anti-tumor activity. Dexamethasone has also been shown to lead to a reduction of T cell proliferation (35). Dexamethasone has also been shown to dampen patients’ immune responses to immune checkpoint blockade (36).

As previously mentioned, the standard of care involves the use of the chemotherapeutic drug temozolomide (TMZ), which is known to influence the immune system. High dose temozolomide induces lymphopenia, an issue that is exacerbated when TMZ is combined with radiotherapy (37). TMZ has also been shown to result in T and B cell dysfunction in a murine model of GBM (38).

In the GBM setting, radiotherapy can be administered in a variety of ways such as whole brain radiotherapy, stereotactic radiosurgery, image guided radiotherapy and hypofractinated radiotherapy (39). Radiotherapy is known to have a number immune modulating effects (40–42), importantly brain tumor exposure to radiotherapy has been shown to upregulate MHC class I expression by brain tumors, and this improves the antigen presentation capability of these cells. Radiotherapy also increases the repertoire of peptides presented by tumor cells and the phenomenon of antigen spreading can occur – i.e. tumor cells die, and their antigens are taken up by nearby immune cells (43). Research has shown that radiotherapy is less efficient in mice lacking T cells, thereby highlighting the additive effect that radiotherapy has in immune cell-mediated control of cancer (44). Radiotherapy is often thought of as an *in-situ* vaccination that makes tumors susceptible to immune attack (44–46). Although a large amount of evidence points towards radiotherapy stimulating an anti-tumor immune response, radiotherapy can also unfortunately result in the secretion of immunosuppressive cytokines such as IL-6 and IL-10 from treated tumor cells (47, 48).

Combined TMZ, radiotherapy and dexamethasone therapy in GBM patients has been shown to induce a persistent lowering of CD4^+^ cell counts which is associated with increased rates of infection and poorer survival (49).

## Immune Inhibitory Proteins Expressed by GBM Tumors

**Figure 1:**
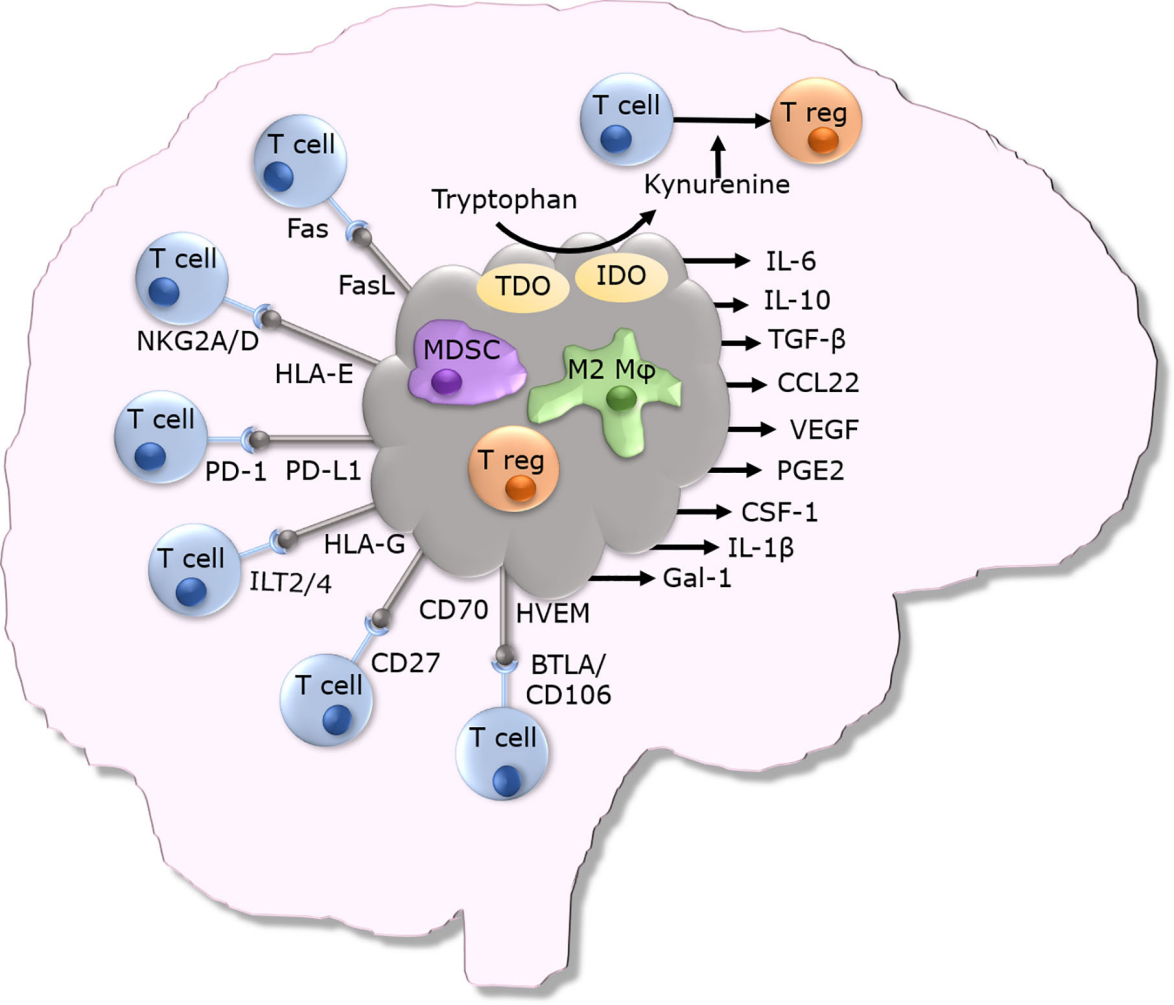
Overview of immunosuppressive mechanisms utilized by GBM tumors.

GBM cells secrete many immunosuppressive proteins and express many cell surface and cytoplasmic immune inhibitory proteins (as summarized in **Figure 1**). Intracellular adhesion molecule 1 (ICAM-1), a key regulator of cell-cell interactions, is commonly upregulated within GBM tumors, when compared to immunohistochemically stained normal brain (50). ICAM-1 interacts with lymphocyte function-associated antigen 1 (LFA-1) expressed on myeloid cells to promote migration of these cells into tumors, thereby enhancing intratumoral immune suppression (51). Myeloid derived suppressor cell (MDSC) accumulation in GBM tumors further contributes to local immune suppression (52). The presence of MDSCs circulating in the blood of GBM patients is also elevated when compared to non-diseased individuals (53). These MDSCs express many immunosuppressive molecules that suppress anti-tumor T cells such as TGF-β and arginase (52). GBM cells have been shown to overexpress galectin-1 (Gal-1), another protein important for the maintenance of cell-cell interactions. Expression of Gal-1 by GBM cells promotes the proliferation and migration of tumor cells (54, 55). Gal-1 expressing GBM cells have also been shown to induce T cell death when the two types of cells are co-cultured (55). Gal-1 interacts with CD45 and CD43 on T cells resulting in their clustering. Gal-1 also binds to CD7 on the T cells and these interactions result in T cell death (56–58).

GBM cells have also been shown to express non-classical MHC class I molecules on their surface which enables them to evade immune cell mediated killing. HLA-G is one such non-classical MHC class I molecule that is involved in immunogenic tolerance of trophoblasts and prevents immune response to the developing semi-allogeneic fetus. In the adult, HLA-G is expressed in thymic epithelial cells, nail matrix and cornea (59). Although HLA-G expression is tightly controlled in the human body, it appears that GBM cells can express HLA-G (59). HLA-G is not just expressed on the cell surface - a soluble isoform that is secreted has been detected in plasma, cerebrospinal fluid and seminal plasma. GBM tumors are frequently infected with cytomegalovirus (hCMV), and hCMV infection has been associated with high levels of HLA-G expression (60). Cytomegalovirus infection is prevalent in the population and infection is lifelong. The immunosuppression linked with GBM results in reduced control of hCMV and this results in reactivation of the virus (60). HLA-G can bind to several receptors, namely the inhibitory receptors Ig-Like Transcript 2 (ILT2) and Ig-Like Transcript 4 (ILT4) (61). HLA-G can also bind the non-inhibitory receptors CD8, CD160, and KIR2DL4. Binding of soluble HLA-G to CD8 on T cells induces a signaling cascade that results in Fas-FasL mediated apoptosis of CD8^+^ T cells (61). HLA-G binding to ILT2 on natural killer (NK) cells inhibits the polarization of lytic granules and the microtubule-organizing center at the contact zone, ultimately preventing NK cell-mediated lysis (61). HLA-E is another non-classical MHC class I molecule; it is a ligand for both NKG2A and NKG2C expressed on NK cells, CD8^+^ αβ and γδ T cells. Binding of HLA-E to NKG2C can lead to immune cell activation, and its binding to NKG2A leads to immune cell inhibition. HLA-E, much like HLA-G, is believed to play a role in maternal tolerance of the fetus (62). HLA-E has been shown to be expressed on GBM cells and this HLA-E expression has been shown to prevent NK cell mediated lysis of these tumor cells. Blockade of the NKG2A – HLA-E interaction has been shown to improve NK cell mediated killing of GBM tumor cells (62).

GBM tumors have also been shown to express Fas ligand (CD95L) on their surface, the binding of which to Fas (CD95/APO-1) on T cells leads to apoptosis of the T cells, thereby enabling GBM cells to evade lysis by Fas-expressing T cells (63). GBMs can also induce T cell death via their expression of CD70. CD70 on GBM cells binds to CD27 on T cells inducing death of activated T cells, and blockade of this interaction has been shown to partially protect T cells from GBM cell induced death (64). GBMs have also been shown to express the immune dampening checkpoint ligand programmed death ligand 1 (PD-L1). PD-L1 binds to its cognate receptor programmed death 1 (PD-1) expressed on activated T cells, and this leads to inhibition of T cell responses to PD-L1 expressing GBM cells. It has been reported that as many as 88% of patient GBM samples express PD-L1 (65). This high level of PD-L1 expression has been shown to be linked with poorer patient survival (66).

Herpes virus entry mediator (HVEM) is an example of another immune checkpoint molecule that has been proven to be expressed in the GBM microenvironment (67). HVEM is usually expressed on T cells, it can have both co-stimulatory and inhibitory effects, depending upon its binding partner (67). HVEM exerts an immune inhibitory effect when bound to B and T lymphocyte attenuator (BTLA) or CD160 expressed by other immune cells (67). High expression of HVEM in GBM tumors has been linked to regulatory T cell differentiation, negatively associated with the regulation of T cell mediated cytotoxicity and with a decreased survival time (67).

Indoleamine 2,3-dioxygenase (IDO) is another protein involved in immunoregulation and prevention of fetal rejection. IDO catabolizes tryptophan into immune-regulatory kynurenines. IDO expression can be induced by a variety of receptors such as the toll like receptors (TLRs), tumor necrosis factor receptor superfamily members (TNFRs), interferon gamma receptors (IFNGRs), transforming growth factor beta receptors (TGFBRs) and aryl hydrocarbon receptors (AhRs) (68). The depletion of tryptophan by IDO activity inhibits immune cell function and prevents dendritic cell (DC) maturation (68). IDO expression is upregulated in recurrent GBMs, with 100% of patients being studied expressing IDO at the time of the second surgery (69). The expression of IDO within GBM tumors is associated with an increased infiltration of CD4^+^ regulatory T cells, immune escape and a poorer prognosis (70). Increased kynurenine production driven by IDO activity induces the differentiation of naïve CD4^+^ T cells into immunosuppressive regulatory CD4^+^ T cells triggered by the binding of kynurenine to the aryl hydrocarbon receptor (AHR) on naïve CD4^+^ T cells (71). Tryptophan 2,3-dioxygenase (TDO), another enzyme involved in the degradation of tryptophan into kynurenine, can also contribute to an immunosuppressive microenvironment high in kynurenine. TDO is expressed in brain tumors and represents a druggable target for reversing the immunosuppressive microenvironment (72).

GBM tumors also secrete numerous other immunosuppressive factors that shape the immune TME and enable immune evasion. GBM tumors secrete IL-6 (73, 74) and their expression of the IL-6 receptor is upregulated (75). IL-6 mediates signaling via the transcription factor STAT3. Upon activation, STAT3 is phosphorylated and persistent phosphorylation is linked with brain tumor grade; with GBM showing the highest levels of STAT3 phosphorylation. Knockdown of STAT3 in GBM cell lines slows *in vitro* and *in vivo* tumor cell growth (76). Human GBM cells isolated from tumors were shown to secrete the chemokine CCL22 (77) which attracts regulatory CD4^+^ CD25^+^ FoxP3^+^ T cells to the TME (78). GBM tumor cells also secrete the immunosuppressive cytokine TGF-β which reduces ICAM-1 and VCAM-1 expression on GBM endothelial cells and thereby T cell infiltration (79, 80). The active form of TGF-β secreted by GBM cells increases the activity of MMP2 and MMP9 on the surface of GBM cells which in turn increases cell motility and promotes the invasion of GBM cells into the surrounding brain (81). GBM tumor cells also secrete the anti-inflammatory cytokine IL-10 which, in the normal setting prevents excessive inflammation and reduces tissue damage by suppressing the activity of Th1 and CD8^+^ T cells. Immune cells such as regulatory T cells secrete IL-10 to quell the immune response (82). IL-10 mRNA is highly expressed in GBM tissues (83). More concerning is that IL-10 not only suppresses the immune system, but also increases the proliferation and migration of GBM cells. Intratumoral microglia/macrophages are major contributors to the IL-10 production within GBM tumors (84).

Human cytomegalovirus (hCMV) is a herpes virus that has been shown to persistently infect 50% to 90% of the adult population. Analysis of GBM tumors has revealed that a large proportion of tumors express hCMV proteins indicating the presence of hCMV within these tumors (85, 86). Human cytomegalovirus secretes a homolog of IL-10, known as cmvIL-10 which has the same immunoinhibitory properties as human IL-10 (87). The attenuation of the immune response by cmvIL-10 prevents eradication of the tumor as well as the virus itself. The secretion of cmvIL-10 leads to the differentiation of CD14^+^ monocytes to macrophages, thereby further supporting hCMV infection (88). *In vitro* studies have revealed that cmvIL-10 affects the maturation and life span of DCs, in that although monocytic DCs exposed to cmvIL-10 reach maturation, their cytokine production is impaired in a non-reversible manner (88). The presence of IL-10 and TGF-β in GBM tumors is believed to downregulate the expression of MHC class I in the TME (89). GBM cells express macrophage migration inhibitory factor (MIF) which renders GBM cells resistant to NK cell mediated killing (90). VEGF secretion by GBM cells stimulates the growth of new blood vessels supplying oxygen and nutrients to rapidly dividing and often hypoxic tumor cells (91, 92). As well as increasing tumor vasculature, VEGF also upregulates expression of the macromolecules tenascin C (TNC) and periostin. TNC blocks the migration of T cells across the blood tumor barrier thereby preventing them from penetrating the tumor parenchyma (23). Periostin also recruits circulating immunoinhibitory M2 macrophages into the tumor parenchyma (93). GBM stem cells secrete the macrophage attracting cytokine periostin. These macrophages support tumor growth and result in a poorer prognosis (93). GBM cells exposed to radiotherapy and chemotherapy have been shown to display increased immunosuppression. This phenomenon has been shown to be due to increased prostaglandin E2 secretion by cells. Blockade of this secreted PGE2 reverses the immunosuppressive capacity of treated cells (47). Colony stimulating factor 1 (CSF-1) is a growth factor that has been shown to be expressed in GBM tumors and by GBM cell lines (94). CSF-1 can either be secreted by cells or expressed as a transmembrane variant on the cell surface. CSF-1 is secreted by astrocytes within the brain during acute inflammatory responses. CSF-1 can bind to its receptor (CSF-1R) on the surface of macrophages and microglia within the brain promoting their switch to the immunosuppressive M2 phenotype (94, 95). GBM cells also secrete interleukin-1α and -1β (IL-1 α and β) (96). The down regulation of HLA class II expression on the U-105 MG GBM cell line by IL-1β suggests that this could be another mechanism which reduces immune recognition by CD4^+^ T cells (97).

## The Contribution of Immune Cells Within GBM Tumors to the Immune Inhibitory Phenotype

Whilst GBM tumor cells contribute to immunosuppression, the immune cells recruited to the tumor can also exacerbate the immune evasive properties of these tumors. Although immune cells can contribute to tumor control, immunosuppressive populations can also contribute to the immune escape of GBM tumors. Indeed, many of the anti-tumor immune cells recruited to the TME adopt an immunosuppressive phenotype due to the cytokines secreted by the GBM tumors and the unique microenvironment which these tumors create.

Myeloid-derived suppressor cells (MDSCs) can be found within GBM tumors, and these cells contribute to the immunosuppressive phenotype of GBMs (98). MDSCs can be divided into two main types, monocytic and granulocytic. Granulocytic MDSCs are rarely found in GBM tumors, whereas the monocytic subtype are more prevalent (99). Monocytic MDSCs support tumor growth by increasing the recruitment of CD4^+^ regulatory T cells via chemokine release in the TME (100). CD4^+^ regulatory T cells are well known immunosuppressive immune cells that dampen the immune response. When compared to healthy controls, the prevalence of regulatory T cells in the peripheral blood is higher in GBM patients. Of even more relevance is that the prevalence of regulatory T cells in lymphocyte populations infiltrating GBM tumors is significantly greater than that in lymphocyte populations from ‘normal’ brain tissue obtained from seizure patients (101, 102). Although immune cell infiltration is often viewed as a positive prognostic marker, it can also contribute to the pathology of GBM. Lymphocytes entering the tumor have been shown to downregulate costimulatory molecules such as CD28 and CD62L (103). The presence of immunosuppressive regulatory T cells within GBM tumors has been correlated with shorter recurrence-free survival. GBM associated microglia/macrophages, which constitute up to 30% of the GBM tumor bulk are of the immunosuppressive M2 phenotype (103, 104). The expression of PD-L1 by these immunosuppressive M2 cells further contributes to local immunosuppression, as does their secretion of CCL22 which recruits regulatory T cells and MDSCs into the TME (103, 104).

## Overcoming GBM-Driven Immunosuppression

### Active Immunotherapy via Vaccination

Vaccination presents an attractive method for immunotherapeutically targeting GBMs (ongoing trials are detailed in **Tables 1**–**3**). One issue that can arise with peptide vaccinations is that immune escape variants can develop, and tumors can overcome the immune pressure applied to them. This phenomenon has been seen in the case of Rindopepimut, an EGFRvIII-keyhole limpet hemocyanin peptide conjugate. When Rindopepimut was used to treat GBM patients with EGFRvIII positive tumors, their median overall survival was 26 months compared to the 15 months of matched controls. Although vaccination prolonged the overall survival of patients, tumors recurred in a large proportion of these patients. When the recurrent tumors were analyzed immunohistochemically for EGFRvIII expression, 82% of the tumors examined had lost expression of EGFRvIII and the other 18% only displayed EGFRvIII expression in less than 1% of their tumor cells (114). These data suggest that the targeting of a single antigen can lead to the generation of immune escape variants, as a consequence of which multiple antigens need to be employed in the formulation of such vaccines.

**Table 1:** Peptide vaccine trials for glioblastoma.

Trial name ClinicalTrial.gov identifier	Phase	Immune targets	Associated treatments in active arm	Control arm	Sample size	T cell response(CD4/CD8 response details)	Humoral response	Median PFS(months)	Median OS(months)	Primary endpoint	Results
IMA-950 NCT01222221 (105)	I	BCAN, CSPG4, FABP7, IGF2BP3, NLGN4X, NRCAM, PTPRZ1, TNC, MET, BIRC5, HBcAg	None	None	40	Yes (Up to 1.1% specific CD8)	NA	NA (PFS6 = 74.4%)	15.3	Safety and immunological response	Positive
NOA-16 NCT02454634 (106)	I	IDH1R132H	None	None	32	Results pending	Results pending	Results pending	Results pending	Safety	Safe vaccine
GAPVAC NCT02149225 (107)	I	Personalized vaccine	None	None	15	Yes (Up to 0.02% specific CD8)	NA	14.2	29	Safety and immunological response	Safe vaccine, Trend for immunological response
SurVaxM NCT01250470 (108)	I	Survivin (SVN53-67/M57-KLH peptide)	None	None	9	Yes (CD8 response in 78% patients: at least 1% specific CD8)	Yes (88% patients)	17.6	86.6	Safety	Safe vaccine
NCT01621542 (109)	I	WT2725	None	None	21	Yes (interim results: CD8 response in 10% patients)	Results pending	Results pending	Results pending	Safety and immunological response	Safe vaccine
UMIN000003506 (110)	I	Cocktail of WT1 HLA class I and II peptides	None	None	14	Yes (CD8 response in 64% patients: median specific CD8 = 6% of total CD8)	NA	4 (r- GBM)	6.2 (r-GBM)	Safety	Safe vaccine
PERFORMANCE NCT02864368	I	CMV peptide	Temozolomide	None	70	Results pending	Results pending	Results pending	Results pending	Safety and immunological response	Results pending
NeoVax NCT02287428	Ia/Ib/ Ic	Personalized neoantigen vaccine	Temozolomide plus Pembrolizumab	None	56	Results pending	Results pending	Results pending	Results pending	Feasibility and safety	Results pending
NCT03223103	Ia/Ib	Personalized mutation-derived tumor antigens	TTF	None	20	Ongoing	Ongoing	Ongoing	Ongoing	Safety	Ongoing
IMA-950 NCT01920191 (111)	I/II	BCAN, CSPG4, FABP7, IGF2BP3, NLGN4X, NRCAM, PTPRZ1, TNC, MET, BIRC5, HBcAg	Pembrolizumab	None	13	Results pending (interim results: CD8 response in 63.2% patients)	Results pending	Results pending (interim results: PFS9=63%)	Results pending (interim results: OS=19)	Safety and immunological response	Positive
SL-701 NCT02078648 (112)	I/II	IL-13Ra2, EphrinA2, survivin	Stage 1: imiquimod; Stage 2: Bevacizumab	None	74	Results pending (interim results: CD8 response in stage 2 patients)	Results pending	Results pending	Results pending (interim results: 11.0 for stage 1, 11.7 for stage 2)	Safety, ORR, OS12	Results pending
IMA950-106	I/II	BCAN, CSPG4, FABP7, IGF2BP3, NLGN4X, NRCAM, PTPRZ1, TNC, MET, BIRC5, HBcAg	None	None	24	Ongoing	Ongoing	Ongoing	Ongoing	Safety	Ongoing
UCPVax-Glio NCT04280848	I/II	Telomerase (TERT)	None	None	28	Ongoing	Ongoing	Ongoing	Ongoing	Immunological response	Ongoing
VBI-1901 NCT03382977 (113)	I/II	CMV (pp65 and gB antigens)	None	None	38	Ongoing	Ongoing	Ongoing (Interim results: 3.6 in immunological responders - rGBM)	Ongoing (Interim results: 14.0 in immunological responders - rGBM)	Safety	Ongoing
ROSALIE NCT04116658	I/II	TAAs and microbiome-derived peptides (EO2401)	Nivolumab +/- Bevacizumab	None	32	Ongoing (not yet recruiting)	Ongoing (not yet recruiting)	Ongoing (not yet recruiting)	Ongoing (not yet recruiting)	Safety	Ongoing (not yet recruiting)
ACTIVATe NCT00643097 (114)	II	EGFR-vIII	Temozolomide	None	22	NA	Yes (33% patients)	NA (PFS5.5 = 66%)	26.0	PFS and immunological response	Positive
ACT II NCT00643097 (115)	II	EGFR-vIII	None	None	18	NA	Yes (43% patients)	14.2	26.0	PFS and OS	Positive
ACT III NCT00458601 (116)	II	EGFR-vIII	Temozolomide	None	65	NA	Yes (85% patients)	9.2	21.8	PFS5.5	Positive (PFS5.5 = 66%)
ReACT NCT01498328 (117)	II	EGFR-vIII	Bevacizumab	KLH and GM-CSF plus Bevacizumab	36 (vs. control 37)	NA	Yes (89% patients)	NA	NA	PFS6	Positive (trend)
SurVaxM NCT02455557 (118)	II	Survivin: SVN53-67/M57-KLH peptide	Temozolomide	None	63	Pending results	Pending results	Pending results (interim results: 13.9)	Pending (interim results: OS12=93.4%)	PFS6	Positive
WIZARD 201G NCT03149003 (119)	II	WT1	Bevacizumab	Bevacizumab	219	Results pending	Results pending	Results pending	Results pending	Safety and OS	Results pending
SurVaxM NCT04013672 (118)	II	Survivin: SVN53-67/M57-KLH peptide	Pembrolizumab	None	51	Ongoing	Ongoing	Ongoing	Ongoing	PFS6	Ongoing
V-Boost	II	Hydrolyzed GBM antigens and alloantigens	Radiotherapy and Temozolomide	None	20	Ongoing	Ongoing	Ongoing	Ongoing	Antitumor activity	Ongoing
ACT-IV NCT01480479 (120)	III	EGFR-vIII	None	KLH and GM-CSF	369 (vs. control 372)	NA	Yes	NA	20.1	OS	Negative

KLH, keyhole limpet haemocyanin, TTF, Tumor Treating Fields; nd-GBM, newly diagnosed glioblastoma; r-GBM, recurrent glioblastoma; TAA, Tumor Associated Antigen; PFS, progression free survival; OS, overall survival; CMV, Cytomegalovirus; EGFR, Epidermal Growth Factor receptor; WT-1, Wilms’ Tumor 1.

**Table 2:** Dendritic cell vaccine trials for glioblastoma.

Trial name ClinicalTrial.gov identifier	Phase	Immune targets	Associated treatments in active arm	Control	Sample size	T cell response (CD4/CD8 response details)	Humoral response	Median PFS (months)	Median OS (months)	Primary endpoint	Results
PERCELLVAC NCT02709616 NCT02808364 (121)	I	Personalized TAA	None	None	5	Yes (CD4 and CD8 response in 80% patients: up to 3.5% specific CD8)	NA	NA	19	Safety	Positive
ATTAC NCT00639639 (122, 123)	I	CMV pp65	None	None	11	Yes (up to 4.5% specific CD8 in 55% patients)	NA	25.3	41.1	Safety and feasibility	Safe vaccine
NCT03615404	I	CMV RNA	Td + GM-CSF + DI-TMZ	None	10	Ongoing	Ongoing	Ongoing	Ongoing	Safety and feasibility	Ongoing
NCT00612001 (124)	I	Autologous glioma lysate vs. GAA peptides	None	None	34	NA	NA	9.6	34.4 for lysate-DC, 14.4 for GAA-DC	Safety and feasibility	Safe vaccine
NCT00068510 (125)	I	Autologous glioma lysate	None	None	12	Yes (CTL response in 50% patients)	NA	15.5	23.4	Safety and feasibility	Safe vaccine
Rudnick 2020 (126)	I	Autologous glioma lysate	Gliadel	None	28	Yes (CD8 response in 25% patients, no details in %specific CD8)	NA	3.6	32 for nd-GBM, 16.3 for r-GBM	Safety and clinical outcome	Positive
MC1272 NCT01957956 (127)	I	Autologous glioma lysate	Temozolomide	None	20	Results pending	Results pending	Results pending (interim results: 9.7)	Results pending (interim results: 20.5)	Safety and feasibility	Safe vaccine
NCT02010606 (128)	I	Autologous glioma stem like lysate	Temozolomide for nd-GBM Bevacizumab for r-GBM	None	38	Results pending	Results pending	Results pending (interim results: 8.6 For nd-GBM; 3.14 For r-GBM)	Results pending (interim results: 21.1 for nd-GBM; 12.0 For r-GBM)	Safety	Safe vaccine
ICT-107 (129)	I	AIM-2, MAGE1, TRP-2, gp100, HER2, IL-13Ra2	None	None	16	Yes (specific CD8 increase in 31% patients)	NA	16.9	38.4	Immunological response	Positive (trend)
NCT01808820	I	Autologous glioma lysate	Imiquimod	None	20	Results pending	Results pending	Results pending	Results pending	Safety	Results pending
NCT03360708	I	Autologous glioma lysate	None	None	20	Ongoing	Ongoing	Ongoing	Ongoing	Safety	Ongoing
ATL-DC NCT04201873	I	Autologous glioma lysate	Pembrolizumab	ATL-DC plus poly ICLC plus placebo	40	Ongoing	Ongoing	Ongoing	Ongoing	Safety and immunological response	Ongoing
NCT03360708	I	Autologous glioma lysate	None	None	20	Ongoing	Ongoing	Ongoing	Ongoing	Safety and toxicity	Ongoing
NCT00890032	I	BTSC mRNA	None	None	50	Ongoing	Ongoing	Ongoing	Ongoing	Safety, Feasibility and immune response	Ongoing
NCT03914768	I	Genetically modified tumour cells and neoantigens	Cyclophosphamide + Bevacizumab	None	10	Ongoing	Ongoing	Ongoing	Ongoing	Safety, feasibility and OS12	Ongoing
NCT01171469 (130)	I	Allogenic BTSCs	Imiquimod	None	8	Increase in IL-17 expressing CD4 (Th17) cells in stable patients compared to non-stable patients	None	NA	NA	MTD and immune response	Vaccine well tolerated with not MTD reached
DENDR-STEM NCT02820584	I	Allogenic BTSC	None	None	20	Ongoing	Ongoing	Ongoing	Ongoing	Safety and Immune response	Ongoing
ICT-121 NCT02049489 (131)	I	CD133	None	None	20	Immune response detected to CD133 epitopes)	NA	NA	NA	Safety and Feasibility	Vaccine was safe and well tolerated
NCT00846456 (132)	I/II	Autologous glioma stem cells lysate	None	None	7	Yes (100% patients, defined via proliferation assay)	NA	23.1	25.3	Safety	Safe vaccine
16-184-4412 (133)	I/II	Autologous glioma cells	None	None	32	Yes (CD8 response in 13% patients: up to 5.5% specific CD8 of total CD8 T cells)	NA	10.3 (r-GMB) 18.3 (nd-GBM)	18.0 (r-GMB) 30.5 (nd-GBM)	Safety, feasibility, immunological response	Positive for safety and feasibility
NCT04388033	I/II	Autologous glioma cells	Temozolomide	None	10	Ongoing	Ongoing	Ongoing	Ongoing	Safety and PFS6	Ongoing
DEN-STEM NCT03548571	II/III	Autologous glioma stem cells	Temozolomide	TMZ	60	Ongoing	Ongoing	Ongoing	Ongoing	PFS	Ongoing
ADDIT-GLIO NCT02649582 (134)	I/II	WT1	Temozolomide	None	20	Ongoing (interim results: CD4 response correlated with OS)	Results pending	Results pending	Results pending (interim results: 43.7)	OS	Ongoing
NCT03879512	I/II	Autologous tumor lysate	Metronomic cyclophosphamide	None	25	Ongoing	Ongoing	Ongoing	Ongoing	Safety and Feasibility	Ongoing
ICT-107 NCT01280552 (135)	II	AIM-2, MAGE1, TRP-2, gp100, HER2 and IL-13Ra2	Temozolomide	TMZ	81 (vs. control 43)	Yes (CD8 response in 50% patients)	NA	11.2	17.0	OS	Positive (trend for OS and significant for PFS)
ICT-107 NCT01006044 (136)	II	AIM-2, MAGE1, TRP-2, gp100, HER2 and IL-13Ra2	Radiotherapy-Temozolimide + fluorescence-guided surgery	None	27	Yes (11/27 patients displayed tumor specific responses with increased serum cytokine levels)	NA	12.7	23.4	PFS	Safe vaccine
DENDR1 EUDRACT N° 2008-005035-15 (137)	II	Autologous tumor lysate	Radiotherapy-Temozolimide	None	22	No	NA	10.5	20.1	PFS12	Positive (PFS12 = 41%)
Audencel NCT01213407 (138, 139)	II	Autologous tumor-derived peptides	Temozolomide	TMZ	34 (vs. control 42)	NA	NA	6.8	18.8	PFS12	Negative
NCT00323115 (140)	II	Autologous glioma lysate	None	None	10	Trends (CD8 and CD4)	NA	9.5	28	Immunological response	Positive trend
NCT01567202 (141)	II	Autologous glioma stem-like lysate	None	Placebo	22 (vs. control 21)	NA	NA	7.7	13.7	OS and PFS	Positive (trend for PFS) (Second phase of trial in IDH1wt TERTmt subgroups of GBM patients ongoing)
NCT01204684	II	Autologous glioma lysate	None	None	60	Results pending	Results pending	Results pending	Results pending	Immunological response	Results pending
AV-GBM-1 NCT03400917	II	Autologous glioma cells	TAA-pulsed DC vaccine plus GM-CSF	None	55	Results pending	Results pending	Results pending	Results pending	OS	Results pending
ELEVATE NCT02366728	II	CMV pp65	+-/Basiliximab	None	100	Results pending	Results pending	Results pending	Results pending	OS and DC migration	Results pending
I-ATTAC NCT03927222	II	CMV pp65	Temozolomide	None	48	Ongoing	Ongoing	Ongoing	Ongoing	OS	Ongoing
ATTAC-II NCT02465268	II	CMV pp65	Temozolomide	Unpulsed PBMC and saline	120	Ongoing	Ongoing	Ongoing	Ongoing	OS	Ongoing
DERIVe NCT03688178	II	CMV pp65	Varlilumab plus Temozolomide	Unpulsed DCs	112	Ongoing	Ongoing	Ongoing	Ongoing	OS, Safety and T reg depletion	Ongoing
GlioVax NCT03395587 (142)	II	Autologous glioma lysate	DC vaccine plus TMZ	TMZ	136	Ongoing	Ongoing	Ongoing	Ongoing	OS	Ongoing
ADCV01 NCT04115761	II	Autologous glioma lysate	Temozolomide	TMZ	24	Ongoing	Ongoing	Ongoing	Ongoing	PFS12	Ongoing
NCT00576537	II	Autologous tumor lysate	None	None	50	Ongoing	Ongoing	Ongoing	Ongoing	Safety and feasibility	Ongoing
ADCTA-G NCT02772094 (143)	II	Autologous tumor lysate	TMZ + Radiotherapy	None	42	NA	NA	NA	22.9 (median for this trial and Taiwan DOH/MA0910072504)	OS and safety	Positive
Combi G-Vax NCT04523688	II	Autologous tumour lysate	TMZ + radiotherapy	None	28	Ongoing	Ongoing	Ongoing	Ongoing	PFS	Ongoing
STING (ICT-107) NCT02546102	III	AIM-2, MAGE1, TRP-2, gp100, HER2 and IL-13Ra2	None	Autologous PBMCs	Estimated 414 but suspended	NA	NA	NA	NA	Overall survival	Suspended
DCVax-L NCT00045968 (144)	III	Autologous tumor lysate	None	Autologous PBMC	331	NA	Results pending	NA	Results pending (interim results: 23.1)	PFS	Results pending
NCT04277221	III	Autologous tumor lysate	Bevacizumab	Bevacizumab	118	Ongoing	Ongoing	Ongoing	Ongoing	OS	Ongoing

KLH, keyhole limpet haemocyanin, TTF, Tumor Treating Fields; nd-GBM, newly diagnosed glioblastoma; r-GBM, recurrent glioblastoma; TAA, Tumor Associated Antigen; PFS, progression free survival; OS, overall survival; Td, Tetanus toxoid; GM-CSF, Granulocyte Macrophage-Colony Stimulating Factor; DI-TMZ, Dose-Intensified Temozolomide; BTSC, Brain Tumor Stem Cells; CMV, Cytomegalovirus; MTD, maximum tolerated dose.

**Table 3:** Other types of vaccine trials for glioblastoma.

Trial nameClinicalTrial.gov identifier	Phase	Type of vaccine	Immune targets	Associated treatments in active arm	Control	Sample size(evaluable patients)	T cell response(CD4/CD8 response details)	Humoral response	Median PFS(months)	MedianOS(months)	Primary endpoint	Results
NCT04015700	I	DNA	Personalized neoantigen	None	None	6	Ongoing (not yet recruiting)	Ongoing (not yet recruiting)	Ongoing (not yet recruiting)	Ongoing (not yet recruiting)	Safety	Ongoing (not yet recruiting)
VXM01 NCT03750071	I/II	DNA	VEGFR2	Avelumab	None	30	Ongoing	Ongoing	Ongoing	Ongoing	Safety	Ongoing
Gliovac – ERC1671 NCT01903330 (145)	II	Tumor cells	Autologous inactivated tumor cells mixed with tumor cell lysates	Cyclophosphamide plus Bevacizumab	placebo plus bevacizumab	84	Ongoing	Ongoing	Ongoing	Ongoing	OS and PFS	Ongoing
HSPPC-96 NCT00293423 (146)	I	HSPPC-96 -peptides	autologous tumor-derived HSPPC-96	None	None	12	Yes (CD8 response in 92% patients - analyzed via re-stimulation essay)	NA	NA	36.0	Safety	Safe vaccine
HSPPC-96 NCT00905060 (147)	II	HSPPC-96 -peptides	autologous tumor-derived HSPPC-96	Temozolomide	None	46	Results pending	Results pending	17.8	23.8 (44.7 in patients with low PD-L1 expression)	Safety, survival	Results pending
NSPPC-96 NCT00293423 (148)	II	HSPPC-96 -peptides	autologous tumor-derived HSPPC-96	Temozolomide	None	41	NA	NA	19.1	42.6	Safety	Safe vaccine
ALLIANCE NCT01814813 (149)	II	HSPPC-96 -peptides	autologous tumor-derived HSPPC-96	Bevacizumab	Bevacizumab	90	Results pending	Results pending	Results pending	Results pending (interim results : 7.5)	OS	Negative
NCT03018288	II	HSPPC-96 -peptides	autologous tumor-derived HSPPC-96	Temozolomide and pembrolizumab	TMZ and Pembrolizumab plus placebo	108	Ongoing	Ongoing	Ongoing	Ongoing	OS12	Ongoing
NCT03650257	II	HSPPC-96 -peptides	HSPPC-96	Temozolomide	Temozolomide	150	Ongoing	Ongoing	Ongoing	Ongoing	OS12	Ongoing

HSPPC-96, heat-shock protein peptide complex 96; KLH, keyhole limpet haemocyanin; PD-L1, Programmed death-ligand 1; PFS, progression free survival; OS, overall surviva.

IMA950 is one such multi-peptide vaccine that is being investigated in GBM. IMA950 is made up of 9 CD8 specific peptides derived from BCAN, CSPG4, FABP7, IGF2BP3, NRCAM, NLGN4X, PTPRZ1, and TNC as well as two CD4 specific peptides derived from survivin and c-met (150). This multi-peptide vaccine was given in conjunction with the immune boosting adjuvant poly-ICLC to GBM patients in a phase I/II clinical trial (111). This vaccination was well tolerated by patients and induced antigen specific CD8^+^ and CD4^+^ T cell responses (111). The level of response seen in patients varied, and analysis of five tumor samples revealed that no vaccine-specific T cells were present in the TIL population, meaning that there may be issues with the homing of vaccine-induced T cells (111). When samples from the recurrent tumors were tested for expression of the target antigens, no change in the levels of these antigens compared to the pre-vaccination tumor samples was observed, further suggesting that the issues are with T cells not trafficking to the tumor site (111). The ability of tumor cells to present immunogenic epitopes at their surface may also explain the failure of peptide vaccine treatments. Whilst tumors may express the target antigen, they may not present the target epitope on their surface meaning that vaccine generated T cells will not target these tumors. Although vaccination with these peptides generates antigen-specific T cells, there appears to be an issue with the immune TME of these tumors. As a result, the combination of IMA950 vaccination with other modalities, such as immune activating anti-CD27 and anti-PD-1 are being explored in the clinic (111).

A ‘personalized’ peptide vaccination approach has also been explored in the GBM setting. In a phase I clinical trial, GBM patients were treated with a cocktail of manufactured peptides derived from known GBM antigens followed by a vaccination that targeted neoepitopes derived from analysis of the patients’ tumor immunopeptidome and transcriptome (107). Each patient received a vaccine that was tailored to their tumor antigen expression profile. Vaccines were administered with the adjuvants Poly-ICLC and GM-CSF. The cocktail of ‘off the shelf’ peptides known as APVAC1 generated CD8^+^ T cell responses in twelve out of the thirteen patients studied and CD4^+^ T cell responses were found in nine of the thirteen patients studied (107). The neoepitope vaccine known as APVAC2 generated a predominantly CD4^+^ T cell response in eight out of the ten patients evaluated. The overall median overall survival of patients receiving this vaccination regime was 29 months (107). Although these findings are promising, these peptide vaccinations are far from curative. Whilst CD4^+^ and CD8^+^ responses were detected, these were at a relatively low level, with the frequency of antigen specific T cells being below 4 percent for CD4^+^ T cells and 1 percent for CD8^+^ T cells (107). The low frequency of target specific T cells may explain the failure of this therapy to act in a curative manner. Targeting of multiple antigens helps prevent the development of antigen escape variants, however combinatorial methods that enable vaccine-induced T cells to penetrate tumors and overcome the immunosuppressive microenvironment need to be explored.

### Targeting Immune Inhibitory Cells and Cytokines

The contribution of macrophages/microglia to the immunosuppressive TME of GBM and their prevalence within the tumor bulk suggest them to be attractive therapeutic targets for the immunotherapeutic targeting of GBM. As mentioned previously, microglia and macrophages in the TME adopt an immunosuppressive M2 phenotype (103, 104). As also previously mentioned, microglia/macrophages express the CSF-1R and GBM cells secrete CSF-1 resulting in the switching of GBM macrophages/microglia to the immune inhibitory M2 phenotype. The blockade of this CSF-1/CSF-1R interaction presents an attractive approach for preventing the switching of tumor resident macrophages/microglia to the immunoinhibitory M2 phenotype. In this regard, blockade of the CSF-1R with the chemical BLZ945 has been shown to improve survival and reduce tumor development in GBM bearing mice without any visible deleterious side-effects. BLZ945 treatment did not alter macrophage numbers within the implanted tumors but reduced the polarization of these macrophages to the M2 phenotype (95). As a result, combining BLZ945 with active immunotherapy represents an exciting therapeutic option for GBM.

As previously discussed GBM cells are known to overexpress MIF, making them resistant to NK cell mediated killing (90). Not only does MIF protect GBM cells from NK cell mediated killing it also exerts effects on macrophages/microglia within the tumors. MIF has been shown to interact with CD74 on microglia resulting in the adoption of the immunosuppressive M2 phenotype. Disruption of the CD74/MIF pathway prevents this M2 phenotype switch and prolongs the survival of GBM tumor bearing mice (151). Immunotoxins have also been used to target tumor associated macrophages (TAMs); Nagai et al. (2009) utilized this methodology to selectively target TAMs. Activated TAMs were shown to express folate receptor beta (FRβ), thereby providing a macrophage-specific target. The heavy and light chains of an anti-FRβ antibody were conjugated to the toxin *Pseudomonas exotoxin* (152). The abundance of macrophages within the tumor allows delivery of the toxin to the tumor resulting in the death of tumor cells and the potentially immunosuppressive macrophages. Administration of this immunotoxin intratumorally to a subcutaneous rat C6 glioma tumor reduced tumor growth and the number of TAMs in these tumors (152). It is important to note that this treatment was injected directly into subcutaneous tumors which reduces the potential for any deleterious off-target effects. Although FRβ was not detected in the normal brain, it was detected on macrophages resident in the heart and liver (152). This presents a potential hurdle to the systemic delivery of this immunoconjugate. The ability of this drug to cross the blood brain barrier is also unknown since this study utilized a subcutaneous model. In patients, this immunoconjugate could be administered intratumorally during surgery, or intraventricularly utilizing an Ommaya reservoir (an intracranial catheter device that allows direct delivery of drugs to the ventricles), thereby bypassing the blood brain barrier. However, this method is highly invasive and not without risks (153, 154).

Propentofylline (PPF) is a synthetic methylxanthine drug that is known to reduce the proliferation (155) and expression of inflammatory cytokines (155) by microglia in response to lipopolysaccharide. PPF could therefore be a novel therapeutic for targeting microglia within GBM tumors. In a rat model of GBM utilizing the CNS-1 cell line, a cell line which recapitulates the features of human GBM with minimal immunogenicity, systemic PPF administration reduced the volume of intracranial CNS-1 tumors (156). *In vitro* analysis revealed that PPF did not exert its effects on the CSF-1 cell line, rather its anti-tumor effects were attributed to its effect on microglial migration and the contribution of microglia to tumor cell migration (156). Rather than trying to remove microglia/macrophages from the TME, switching immunosuppressive M2 cells to the immune activating M1 phenotype also represents an attractive therapeutic option.

IL-12 represents an excellent immunotherapeutic candidate due to its ability to activate T-cells and NK cells and provoke antigen-specific immunity (157). As systemic administration of recombinant IL-12 was associated with adverse effects (such as damage to vital organs), gene transfer of IL-12 was achieved by the intracranial administration of an adeno-associated virus (AAV) encoding IL-12 to rats, after which they were challenged by intracranial injection of rat RG2 GBM cells. Treatment improved the survival of tumor challenged mice when compared to PBS injected control mice. Analysis of treated tumors revealed an increase in the microglial activation markers ED1 and TNF-related apoptosis-inducing ligand (TRAIL), and this was accompanied by a downregulation of the proliferation marker Ki67 and an increase in TUNEL staining - an indicator of apoptosis (157).

The blockade of TGF-β presents an attractive adjunct for active immunotherapy, due to its immunoregulating and tumor promoting effects. Trabedersen is an anti-sense RNA for human TGF-β2 mRNA that has been administered via convection-enhanced delivery to patients with recurrent GBM. Although Trabedersen improved the median survival compared to chemotherapy alone, this difference was not of statistical significance (158). In a pre-clinical murine model of metastatic pancreatic cancer, active vaccination was combined with antibody blockade of TGF-β. Soares et al. (2015) treated a murine model of pancreatic cancer using a vaccine comprised of GM-CSF secreting irradiated pancreatic cancer cells known as GVAX. This vaccine was used to treat two models of pancreatic cancer, the Panc02 model and KPC model. When GVAX vaccination was combined with TGF-β blockade, the cure rate of tumor bearing mice was improved in both models when compared to mice given GVAX with an IgG isotype antibody. The anti-tumor effects of GVAX were even further improved when the vaccine was combined with both an anti-TGF-β and anti-PD-1 antibody. This blockade of TGF-β in combination with GVAX reduced the regulatory T cell infiltrate into these tumors, a trend not seen when either therapy was used alone (159).

### Immune Checkpoint Blockade

Due to the expression of numerous immunosuppressive checkpoints within the GBM TME, many checkpoint blockade antibodies have been tested in the GBM setting. Immune checkpoint blockade also represents a method for rescuing exhausted T cells. As monotherapies, immune checkpoints have provided lackluster results (160–162). One interesting method for altering the responsiveness to immune checkpoint blockade is to administer these immune checkpoint blocking antibodies in a neoadjuvant setting, as opposed to an adjuvant setting. Neoadjuvant administration of checkpoint blockade involves the dosing of the patient prior to tumor resection and standard therapy as opposed to after surgery and alongside standard therapy. In the GBM setting, neoadjuvant PD-1 blockade has been explored patients with recurrent disease - these patients received neoadjuvant PD-1 and therapy was then continued in the adjuvant setting post-surgery. Neoadjuvant treatment prolonged the overall survival when compared to adjuvant PD-1 blockade, and increased CD8^+^ T cell infiltration into tumors. An upregulation in the expression of interferon gamma related genes was also seen in the tumors of these patients (163).

Combining checkpoint blockade modalities or combining active immunotherapy with checkpoint blockade are also attractive methods for enhancing protective anti-GBM immunity. In a pre-clinical murine model of GBM, PD-1 blockade was combined with DC vaccination to great effect. Mice bearing intracranial GL261 tumors were vaccinated with DCs loaded with murine GL261 tumor cell lysate. Although this approach increased the infiltration of tumor cells into these intracranial tumors, this did not lead to improved survival in mice with an elevated tumor burden. It was hypothesized that local immune suppression within the TME was preventing tumor-specific lymphocytes from inducing tumor cell death. TILs were shown to have up-regulated their expression of PD-1, as a result of which it was decided to combine anti-PD-1 antibody therapy with DC vaccination. This combination increased the percentage of activated CD8^+^ T cells within the intracranial tumors and improved the survival of mice when compared to mice given vaccination alone (164).

As CSF-1R inhibition has been shown to reduce polarization of macrophages to the immunosuppressive M2 phenotype (95), combining CSF-1R inhibition with active vaccination and PD-1 blockade has been explored in the GBM setting. Myeloid derived cells recruited to the tumor were shown to express PD-L1 and contribute to the immunosuppressive environment seen in murine GL261 tumors. The presence of vaccine-induced TILs increased the recruitment of these immunosuppressive PD-L1 expressing myeloid cells. As a result, Antonios et al. (2017) combined PD-1 antibody and a CSF-1R inhibitor with active DC vaccination. CSF-1R inhibition increased the presence of TILs within tumors, whereas PD-1 blockade improved the activation of TILs. This triple therapy significantly increased the survival of GL261 tumor bearing mice when compared to non-treated, DC vaccinated and DC vaccinated mice with either CSF-1R or PD-1 blockade alone (165).

As detailed earlier, IDO and TDO expression within GBM tumors contributes to the immunosuppressive nature of these tumors. Targeting IDO alone or as part of a combinatorial strategy therefore also represents an attractive treatment avenue. The anti-viral drug acyclovir has been shown to inhibit both IDO and TDO and preventing the recruitment of regulatory T cells to the TME (166). In a pre-clinical murine model of GBM, the combined blockade of IDO, CTLA-4 and PD-1 reduced regulatory T cell infiltration into tumors and led to 100% long-term survival in mice harboring intracranial GL261 tumors (167).

### Engineered CAR T Cells

Chimeric antigen receptor (CAR) T cells provide an avenue for generating tumor targeted T cells that can function in the defective tumor microenvironment. CAR T cells are generated by transfecting autologous T cells taken from patients with a construct combining a single chain variable fragment specific to a tumor cell target with costimulatory domains that enable T cell activation without the need for a secondary co-stimulatory signal (168). Numerous antigens have been targeted utilizing CAR T cells and the design of CAR T cells has been fine-tuned in order to optimize their anti-tumor activity. Traditionally CAR T cells’ intracellular signaling domain was derived from the CD3ζ chain of the T cell receptor (first generation), as progress has been made further costimulatory domains have been added to the intracellular region in order to improve the functionality of CAR T cells (second and third generation). These costimulatory domains are often derived from costimulatory CD28, OX-40, ICOS, and 4-1BB (169, 170). Whilst the design of the targeting domain of the CAR T cells has evolved so has the general design of these cells, with the knock in of other genes that enhance anti-tumor function being explored (see **Table 4**). As mentioned previously, GBM tumors frequently upregulate their expression of FasL (63, 175). CAR T cells generated from patient derived T cells often express Fas, which makes these T cells susceptible to FasL mediated cytotoxicity when entering the TME (176). The development of CAR T cells expressing Fas dominant negative receptors by Yamamoto and colleagues resulted in the persistence of cells without any deleterious side-effects such as autoimmunity or lymphoproliferative disease (177). CAR T cells expressing a dominant negative receptor for TGF-β have also been developed for the treatment of prostate cancer. These CAR T cells target a prostate antigen known as prostate-specific membrane antigen (PSMA) and they also express the dominant negative TGF-βRII that blocks TGF-β signaling. These CAR T cells displayed improved anti-tumor function when compared to CAR T cells that did not have the dominant negative TGF-βRII transfected into them. These CAR T cells appeared to exhibit long-term persistence and resistance to exhaustion (178). CAR T cells have also been engineered to secrete a PD-1 blocking antibody single chain variable fragment (scFv) that binds to PD-1 on the surface of activated T cells (both CAR and bystander T cells), thereby preventing PD-L1 on tumor cells from dampening T cell anti-tumor responses (179). These CAR T cells enhance the survival of PD-L1 expressing tumor bearing mice when compared to CAR T cells that do not secrete the PD-1 scFv combined with an anti-PD-1 antibody. This is believed to be due to the increased amount of PD-1 blockade within the TME when compared to systemic checkpoint blockade. These CAR T cells displayed efficacy against both hematologic and solid tumors (179). CAR T cells have also been modified to express the immune-stimulatory molecule CD40L to improve the anti-tumor function of these cells (180). The interaction of CD40L on these T-cells with CD40 on DCs results in the secretion of the immunostimulatory cytokine IL-12 (180). CD19 directed CAR T cells armed with the CD40 ligand have been shown to lyse CD19 negative cells and prevent their expansion and the development of antigen negative variants that escape an immune response (180). In order to prevent the development of antigen escape variants, CAR T cells have also been developed to produce bi-specific T cell engagers (BiTEs) in the GBM setting. EGFRvIII targeting CAR T cells have been developed to secrete BiTEs that target the wild type epidermal growth factor receptor (EGFR). These BiTEs contain an anti-EGFR domain along with an anti-CD3 domain, homing T cells onto EGFR expressing tumor cells. The secretion of these BiTEs recruits bystander cells that target tumor cells, these CAR T cells can also eradicate tumors that do not express the EGFRvIII antigen, thereby highlighting the importance of the BiTEs produced by these CAR T cells (181). One study looked at utilizing CD123 (IL-3 Receptor α chain) directed CAR T cells to target Hodgkin lymphoma cells. The investigators also hypothesized that as CD123 is expressed on myeloid cells, these CAR T cells could also target these cells and overcome the local immune suppression induced by MDSCs and M2 macrophages. These CAR T cells targeted lymphoma cells *in vitro* and *in vivo*. What was even more interesting was that these CAR T cells were resistant to inhibition by M2 macrophages when compared to classical CD19 targeting CAR T cells (182).

**Table 4:** CAR T cell trials for glioblastoma.

Trial name ClinicalTrial.gov identifier	Phase	CAR generation	Targets	Associated treatments in active arm	Sample size(evaluable patients)	Median PFS(months)	MedianOS(months)	Primary endpoint	Results
NCT01109095 (171)	I	Second	HER2 and CMV pp65	None	16	NA	24.8 months for children and 30 months for adults	Safety and feasibility	Positive
NCT02442297	I	Second	HER2	None	28	Ongoing	Ongoing	Safety and feasibility	Ongoing
NCT02208362 (172)	I	Second	IL13Rα2	None	92	Ongoing	Ongoing	Safety and feasibility	Ongoing
NCT02209376 (173)	I	Unknown	EGFRvIII	None	10	Not evaluable	8 months	Safety and feasibility	CAR T cells seen to traffic to tumours, however adaptive changes in TME need to be accounted for
NCT02664363	I	Third	EGFRvIII	TMZ induced lymphodepletion	3	Ongoing	Ongoing	MTD	Ongoing
NCT04003649	I	Second	IL-13Rα2	Ipilimumab and Nivolumab	60	Ongoing	Ongoing	Safety and feasibility	Ongoing
INTERCEPT NCT03283631	I	Unknown	EGFRvIII	None	24	Ongoing	Ongoing	MTD	Ongoing
NCT02844062	I	Unknown	EGFRvIII	None	20	Ongoing	Ongoing	Saftey and feasibility	Ongoing
NCT03726515 (174)	I	Unknown	EGFRvIII	Pembrolizumab	7	Ongoing	Ongoing	Safety, feasibility, OS and PFS	Ongoing
NCT04077866	I	Unknown	B7-H3	TMZ	40	Ongoing	Ongoing	OS	Ongoing
NCT04045847	I	Unknown	CD147	None	31	Ongoing	Ongoing	Safety and feasibility	Ongoing
NCT02937844	I	Second	PD-L1 (PD-1 on CAR T cell linked to co-stimulatory CD28 cytoplasmic domain)	Cyclophosphamide and Fludarabine	20	Ongoing	Ongoing	Safety and feasibility	Ongoing
NCT04270461	I	Third	NKG2D	None	10	Ongoing	Ongoing	Safety and feasibility	Ongoing
NCT04385173	I	Unknown	B7-H3	TMZ	12	Ongoing	Ongoing	Safety, feasibility, OS and PFS	Ongoing
NCT01454596	I/II	Third	EGFRvIII	Chemotherapy induced lumphodepletion and aldesleukin	18	Ongoing	Ongoing	Safety, feasibility and PFS6	Ongoing

PFS, progression free survival; OS, overall survival; MTD, maximum tolerated dose.

### Oncolytic Virotherapy

The design and delivery of immunotherapies must consider the pronounced immunosuppressive environment of the TME in GBM. The use of oncolytic viruses, which can selectively infect and kill tumor cells, is beginning to generate increased interest due to its tumor specificity and the ability of these viruses to turn an immunosuppressive microenvironment into an immune supporting environment (183). Oncolytic viruses are genetically altered to not infect non-transformed cells, and, in some cases, other genes may be knocked down or knocked in to enhance the immune stimulatory properties of these viruses. For example, the oncolytic herpes simplex virus T-VEC has been transfected with the human *granulocyte-macrophage colony-stimulating factor (GM-CSF)* gene. GM-CSF secreted by the virus increases the recruitment of DCs into the TME and thereby enhances antigen presentation and T cell activation (184). Tumor cell lysis by oncolytic viruses also triggers inflammatory immune responses involving the release of antigens, danger associated molecular patterns (DAMPs) and pathogen associated molecular patterns (PAMPs) within the TME (184). Several different types of viruses have been used in the oncolytic virotherapy of GBM, viruses such as the herpes simplex virus (HSV), Newcastle disease virus (NDV), poliovirus, reovirus, adenovirus, measles virus and H1 parvovirus (185) (see **Table 5**). Not only have viruses been used to directly induce the death of tumor cells they have also been used to transfer genes to tumor cells that enable these cells to be targeted. One such example of one of these viruses is Toca 511, a retroviral vector that delivers cytosine deaminase to rapidly dividing malignant cells (193). The transferred cytosine deaminase enzyme then converts the pro-drug 5-fluorocytosine to the active antineoplastic compound 5-fluorouracil resulting in the death of tumor cells (193). The use of this virus pro-drug combination has also been shown to result in an increase of immune cell activity within murine brain tumors, with a decrease in immunosuppressive cells and an increase in interferon gamma positive CD8 T cells within the tumor microenvironment (194). Whilst in treating preclinical models of GBM Toca 511 showed great promise recent however results from a phase II/III clinical trial revealed that Toca 511 in combination with 5-fluorocytosine did not improve overall survival when compared to standard therapy (195). Although oncolytic viral therapy represents an exciting avenue for GBM therapy, it is not without obstacles and as a result combinatorial therapy utilizing oncolytic viruses needs to be considered. Very little virus crosses the blood brain barrier when oncolytic viruses are delivered systemically, yet these therapies are still efficacious in brain tumor models. Oncolytic herpes simplex viruses (HSVs) can be used in combination with various other therapeutics for the treatment of GBM. The virus can also be altered with immunomodulating transgenes to improve anti-tumor efficacy and enable modulation of the TME (196). ‘Arming’ an oncolytic HSV with the murine IL-4 gene has been shown to increase the survival of mice bearing intracranial GL-261 cells. Conversely, no survival benefit compared to sham treated animals was observed when immunosuppressive IL-10 was transfected into this oncolytic virus (197). Clinical testing of oncolytic viruses remains in its relative infancy, with several viral therapies undergoing phase I/II clinical trials (198). The prospect of genetically modifying these viruses provides great hope for the future treatment of GBM. Not only can viruses be genetically manipulated but they can also be combined with other immunotherapeutic modalities to help overcome the immunosuppressive TME. One such example is combination of oncolytic measles virus therapy with anti-PD-1 checkpoint blockade (199). This combination was shown to increase survival in C57BL/6 mice bearing intracranial GL261 tumors when compared to either monotherapy, as well as increasing survival this combinatorial therapy increased T cell infiltrate into these tumors (199). Checkpoint blockade has also been combined with the IL-12 expressing oncolytic HSV in a pre-clinical model of GBM to great effect (200). This IL-12 secreting HSV was combined with both anti-CTLA-4 and anti-PD-1 checkpoint blockade for the treatment of two murine GBM models, this triple therapy reduced the number of regulatory T cells present within tumors and increased the influx of immune supporting M1 macrophages resulting in the complete cure of these mice (200). As previously mentioned GBMs frequently overexpress PGE2 which promotes an immunosuppressive environment and provides an attractive target for therapy. An oncolytic vaccina virus has been developed that expresses 15-(NAD)-hydroxy-prostaglandin-inactivating enzyme (HPGD); an enzyme that inactivates PGE2 (201). This modified vaccinia virus was tested in a variety of mouse solid tumor models and it was found to reduce the number of MDSCs and regulatory T cells within these tumors increasing the response of these tumors to viral therapy and adoptive T cell transfer (201). Whilst viral therapy is in its relative infancy with regards to clinical approval these early findings provide great hope for the future of this treatment modality.

**Table 5:** Viral therapy trials for glioblastoma.

Trial name ClinicalTrial.gov identifier	Phase	Virus used/mode of action	Associated treatments in active arm	Control	Sample size(evaluable patients)	Median PFS(months)	MedianOS(months)	Primary endpoint	Results
NCT00390299	I	Oncolytic carcinoembryonic antigen expressing measles virus (MV-CEA)	None	None	23	Ongoing	Ongoing	Safety, feasibility OS and PFS	Ongoing
NCT02444546	I	Reovirus (REOLYSIN®)	Sargramostim (GM-CSF)	None	6	Ongoing	Ongoing	MTD and safety	Ongoing
NCT00528684	I	Reovirus (REOLYSIN®)	None	None	18	Ongoing	Ongoing	MTD and safety	Ongoing
NCT00031083	I	Adenoviral transfer of IFN-β gene	None	None	35	Suspended	Suspended	Safety and feasibility	Suspended
NCT03043391	I	Poliovirus (PVSRIPO)	None	None	12	Ongoing	Ongoing	Safety, feasibility and OS24	Ongoing
NCT03072134	I	Neural stem cells loaded with adenovirus	None	None	13	Ongoing	Ongoing	Safety and feasibility	Ongoing
NCT03911388	I	HSV G207	+/- Single dose of 5 Gy radiation	None	15	Ongoing	Ongoing	Safety and feasibility	Ongoing
NCT01491893 (186)	I	Poliovirus (PVSRIPO)	None	Historical controls	15	Results pending	Results pending (interim: 12.6)	MTD, safety and feasibility	Positive
NCT02457845 (187)	I	HSV G207	None	None	5	Results pending	Results pending	Safety and feasibility	Positive
									
D24GBM NCT01956734	I	Adenovirus (DNX-2401)	TMZ	None	31	Pending	Pending	Safety PFS6 and OS12	Pending
NCT02197169 (188)	I	Adenovirus (DNX-2401)	+/- IFNγ	None	27	Results pending	Results pending (interim OS12 = 33 %, interim OS18 = 22 %)	Safety and feasibility	DNX-2401 was well tolerated however the addition of IFNγ made no difference to efficacy
NCT03657576	I	C134-HSV	None	None	24	Ongoing	Ongoing	Safety and efficacy	Ongoing
NCT03152318	I	HSV (RQNestin34.5v.2)	+/- Cyclophosphamide	None	108	Ongoing	Ongoing	MTD	Ongoing
NCT02026271 (189)	I	Ad-RTS-hIL-12	Veledimex	None	31	NA	12.7	Safety and feasibility	Positive
NCT03636477	I	Ad-RTS-hIL-12	Veledimex + Nivolumab	None	21	Ongoing (not recruiting)	Ongoing (not recruiting)	Safety and feasibility	Ongoing (not recruiting)
NCT03896568	I	Allogenic stem cells loaded with adenovirus (DNX-2401)	None	None	36	Ongoing	Ongoing	Safety, feasibility and MTD	Ongoing
NCT03679754	I	Ad-RTS-hIL-12	Veledimex	None	36	Ongoing	Ongoing	Safety and feasibility	Ongoing
NCT01811992	I	Ad-hCMV-TK and Ad-hCMV-Flt3L	None	None	19	Ongoing (not recruiting)	Ongoing (not recruiting)	Safety and feasibility	Ongoing (not recruiting)
NCT03714334	I	DNX-2440	None	None	24	Ongoing	Ongoing	Safety, feasibility and OS	Ongoing
NCT02031965	I	HSV-1716	Dexamethasone + surgery	None	2	Results pending	Results pending	MTD	Results pending
NCT02062827	I	HSV-1	None	None	36	Ongoing	Ongoing	MTD	Ongoing
NCT04327011	I	Toca 511/5-FC	None	None	65	Terminated	Terminated	Safety and OS	Terminated
NCT00028158	I/II	HSV G207	None	None	65	Results pending	Results pending	Safety and feasibility	Results pending
NCT01301430 (190)	I/II	Parovirus H-1 (ParvOryx)	None	None	18	Results pending	Results pending	Safety and feasibility	Results pending
ONCOVIRAC NCT03294486	I/II	TG6002/5-FC	None	None	78	Ongoing	Ongoing	Safety and feasibility	Ongoing
NCT01582516	I/II	Adenovirus (Delta-24-rgd)	None	None	20	Results pending	Results pending	Safety, feasibility and OS	Results pending
NCT00589875 (191)	II	AdV-tk	Valacyclovir + standard of care	Matched control cohort	48	12.7	25.1 for patients with maximal resection	Safety, feasibility and OS	Positive
NCT04482933	II	HSV G207	Single dose of 5 Gy radiation	None	30	Ongoing (Not yet recruiting)	Ongoing (Not yet recruiting)	OS	Ongoing (not yet recruiting)
NCT02798406	II	Adenovirus (DNX-2401)	Pembrolizumab	None	49	Ongoing (not recruiting)	Ongoing (not recruiting)	Objective response rate	Ongoing (not recruiting)
NCT00870181 (192)	II	ADV-TK	Ganciclovir + chemotherapy	None	47	8.7	11.4	PFS6	Positive
NCT04406272	II	VB-111	Bevacizumab	Standard of care	45	Ongoing	Ongoing	TIL density and dose limiting toxicity	Ongoing
NCT04006119	II	Ad-RTS-hIL-2	Cemiplimab	None	36	Ongoing (not recruiting)	Ongoing (not recruiting)	Safety, feasibility and OS	Ongoing (not recruiting)
NCT04105374	II/III	Toca 511/Toca FC	TMZ + radiotherapy	Standard of care	Terminated	Terminated	Terminated	PFS and OS	Terminated

PFS, progression free survival; OS, overall survival; MTD, maximum tolerated dose.

### Combining Immunotherapy With Standard Therapy

Adapting current therapies also needs to be considered in the context of immunotherapy for GBM, especially given the likelihood that all new approaches will need to be delivered in the context of current ‘standard’ therapy. Both TMZ and radiotherapy have immune augmenting effects that can be capitalized upon when considering the immunotherapeutic treatment of GBM. As mentioned previously, TMZ can induce lymphodepletion in patients. This lymphodepletion can be capitalized on to potentially enhance the efficacy of CAR T cell therapy. In a murine model of GBM, EGFRvIII CAR T cells failed to confer a survival advantage for mice bearing intracranial EGFRvIII expressing tumors, despite that fact that these cells were shown to have anti-tumor cell activity *in vitro*. Lymphodepletion with radiotherapy administered prior to CAR T cell therapy was shown to improve the efficacy of CAR T cell therapy by resulting in long-term survival of mice (202). Similarly, TMZ was used to lymphodeplete prior to CAR T cell administration. TMZ was either used in a standard or high dose, with the higher dose inducing more marked lymphodepletion. The lymphodepletion caused by high dose TMZ increased the survival of mice bearing established intracranial tumors when given CAR T cell therapy. This lymphodepletion to led to persistence of the injected CAR T cells within the blood of treated mice and this correlated with lower tumor burden (202). As well as using high dose TMZ to lymphodeplete, the dosing can also be given as low frequent doses, known as metronomic dosing. Ouyang and colleagues (2016) designed immune activating CpG carbon nanotube conjugates (SWCNT/CpG-2) that prolonged the survival of mice bearing intracranial GL261 tumors. This SWCNT/CpG-2 was used to treat a more invasive GBM model using the KR158B cell line, a model that more faithfully represents the characteristics of human GBM within a murine model. Although this intracranial SWCT/CpG-2 therapy was not curative for this KR158B model, as it was in the case of the GL261 model, when this SWCT/CpG-2 was combined with low dose daily TMZ, it significantly improved survival when compared to SWCT/CpG-2 monotherapy (203). Splenocytes taken from mice that had received metronomic TMZ in combination with SWCT/CpG-2 were more efficient at inducing *in vitro* KR158B tumor cell death than splenocytes from mice given either SWCT/CpG-2 or TMZ alone. This dual therapy did not reduce the number of regulatory T cells in the tumors. However, both SWCT/CpG-2 therapy and dual therapy induced an increased macrophage infiltrate into the tumors. The researchers hypothesized that the metronomic TMZ dosing increased the relative proportions of immune activating M1 macrophages to immune inhibitory M2 macrophages within the tumors (203). Radiotherapy can also be used as an adjunct to immunotherapy in order to boost the anti-tumor immune response. Weiss *et al*. (2018) generated an NKG2D expressing CAR T cell therapy that when systemically administered penetrated brain tumors in a murine GL261 GBM model. These NKG2D CAR T cells were shown to cure 22% of GL261 bearing mice treated. Radiotherapy upregulated the expression of NKG2D ligands on the surface of GBM cells and, as a result, it was decided to combine radiotherapy with NKG2D CAR T cells. Mice were given a single 4 gray (Gy) dose of radiotherapy on day 7 after tumor implant and CAR T cells were given on days 5, 7 and 10. The single radiotherapy dose alone did not alter the survival of tumor bearing mice compared to control mice, however it increased the survival of mice harboring intracranial GL261 cells when combined with the NKG2D CAR T cell therapy. This effect was also shown in mice bearing intracranial SMA-560 tumor cells (204). Another alternative that has been considered is the intratumoral administration of TMZ, as opposed to systemically administered TMZ. This local delivery of TMZ could theoretically prevent the profound lymphodepletion seen in systemic administration due to the therapy being mainly confined to the tumor. This method of administering TMZ was shown to improve the survival of mice bearing GL261 cell-derived tumors when compared to mice given intraperitoneal TMZ. CD4 and CD8 blocking antibodies revealed that T cells are responsible for this improved survival, with mice receiving intracranial TMZ failing to show improved survival if T cell blocking antibodies were used. Survival was improved even further when intracranial TMZ was combined with active immunotherapy using irradiated GL261 cells transfected to express GM-CSF (205). This combined intracranial TMZ and immunotherapy increased CD8^+^ T cell infiltrate and decreased MDSCs (205).

### Overcoming the Blood Brain Barrier (BBB)

The BBB can act as a significant barrier for systemically administered therapeutics, including immune checkpoint blocking antibodies. Several approaches can be used to address this issue. These include direct modification and masking of therapeutic agents, encapsulation of therapeutics within vesicle-based delivery systems, and targeted opening of the BBB/BTB by physical or biochemical disruption. Conceptually, the simplest route to bypass the BTB is direct administration to the brain parenchyma or the cerebrospinal fluid (CSF). Although this is a commonly used approach in pre-clinical, experimental work, it is clinically problematic. Direct intra-parenchymal injection is rarely performed outside of intensive care medicine due to the difficulties associated with infection risk and needle damage. Moreover, although GBM are rarely metastatic (206), direct administration to the tumor site is contra-indicated due to the slow rate of diffusion of therapeutic molecules through compact brain tissue, injected substances rarely travelling more than a few millimeters beyond the injection site (207–209). This route is therefore unlikely to be sufficient for treating GBM, given both the likely tumor size on diagnosis and accessibility issues. Intracerebroventricular or intrathecal injection, delivery to the CSF, has similarly poor distribution issues (210). Passage of drugs from the CSF to the parenchymal tissue is primarily diffusive, which, coupled with rapid removal from the ependymal surface via bulk flow and the glymphatic system, results in minimal transfer of therapeutic agents into the tissue (211, 212). These restrictions are even more relevant to the delivery of large molecules such as therapeutic antibodies (213).

Working on the principle that the simplest way to overcome the BBB/BTB is to remove it, a number of methods of disrupting barrier function have been investigated for their potential use in the treatment of GBM and other neurological disorders. Such techniques were first begun over 50 years ago, with studies employing hypertonic solutions of osmolytes such as mannitol to induce osmotic endothelial shrinkage and tight junction opening (214). Such untargeted disruption, whilst effective in permitting increased therapeutic access to the brain, is also indiscriminate and enables the entry of pro-inflammatory and potentially toxic serum proteins such as albumin and complement factors (215), thereby rendering this non-specific approach unsuitable for clinical use. More targeted methods of inducing increased BBB/BTB permeability have used endogenous bioactive agents such as bradykinin or its synthetic analogues (216). Although such approaches have increased permeability of chemotherapeutic agents to the brain in preclinical models, they have not translated into clinical practice, possibly due to having too brief a duration of action (217).

Rather than chemical or osmotic-mediated disruption, another technique used to circumvent the BBB/BTB is the use of high-power focused ultrasound (218) to generate foci of increased tissue permeability. Although this approach is effective in opening the barrier to therapeutic antibodies (219), it suffers from producing bystander tissue distortion and damage in experimental animals (220). In an attempt to overcome this issue, the technique has been refined to improve specificity and reduce energy transfer through the use of injected microbubbles (221, 222). In this case, lower frequency ultrasound is used to stimulate microbubble oscillation and cavitation, disrupting the endothelial wall through local shock wave production and permitting access of therapeutic agents to the brain. Although promising, it is not yet clear to what degree brain penetration can be enhanced as efflux transport systems remain active (223), and the long-term consequences of disruption have not yet been studied.

As an alternative to BBB/BTB disruption, numerous attempts have been made to modify the therapeutic agents themselves or their delivery systems to permit greater transfer across an intact BBB/BTB. Building on the rationale that more lipophilic agents are better able to cross the BBB/BTB, initial approaches aimed to improve therapeutic agent lipid solubility. Although such modifications do indeed improve CNS access, this was achieved at the cost of increased non-specific membrane permeability and a consequent rise in off-target effects (224, 225).

To overcome these difficulties, ongoing attempts at achieving effective drug delivery across the BBB/BTB have employed a wide range of different nanocarriers, also termed nanoparticles. These are diverse molecular structures, including lipid micelles, liposome composites of phospholipid and other molecules, and polymer-based particles, with the common property that they form a vesicle that can be loaded with therapeutic agents and which can then cross the BBB to enter the parenchyma (226). Once within the brain, variation in environmental pH at the tumor site, amongst other conditions destabilize the nanocarrier structure and trigger release of the cargo within the tissue (227). Although effective, these nanocarriers are indiscriminate and passively deliver their cargo widely across the brain, a drawback that has spurred the development of more effectively targeted nanocarrier delivery systems.

Targeting can be substantially enhanced by including molecular tags within the vesicle wall using proteins, peptides, nucleic acids or small molecules that specifically recognize tumor-associated receptors, thereby minimizing off-target actions. A wide range of different molecular tags have been exploited for this purpose *in vivo*, including for example, the interaction of nanocarrier borne transferrin with the transferrin receptor TfR-1 on GBM cells (228), the EGFP-EGF1 fusion protein on nanoparticles with tissue factor in tumor cells (229), and cholera toxin with tumor-expressed chloride channels and matrix metalloproteinase-2 (230). Such strategies hold significant promise as they allow for both the concentration of therapeutic agents at the tumor site and, by virtue of the encapsulation, protect therapeutic agents from hepatic metabolism (230). Although a number of nanocarrier-encapsulated small molecule approaches are currently undergoing clinical trial in GBM, as yet none have been approved for use (231). Questions about the efficiency of large molecule, i.e. therapeutic antibody, encapsulation efficiency remain.

As direct lipophilic modification of therapeutic agents and encapsulation strategies have relatively broad specificity, even with improved targeting strategies, interest has grown in the use of direct molecular tagging of the therapies themselves to permit recognition by specific endothelial transporters, e.g. the transferrin receptor, insulin receptor or low density lipoprotein receptor (232, 233), a process sometimes termed receptor mediated transcytosis.

This approach has proven to hold significant promise for the experimental delivery of protein agents, including therapeutic antibodies. Exposure of ‘normal’ CNS to circulating biologic agents is restricted to less than 0.5% of the concentrations that are present in serum (234, 235), a level at which target engagement is unlikely to occur (236). However, molecular engineering of therapeutic antibodies has enabled significant enhancements in uptake across the BBB. These approaches include the development of bispecific antibodies in which one F(ab) binds the target of interest and the other binds and is transported by an endothelial transporter (237), therapeutic antibodies in which a transporter recognition domain is linked to the immunoglobulin heavy or light chain (238, 239), or, more recently, molecules in which the Fc domain itself is directly recognized by an endothelial transporter (240, 241). Such an approach has not yet been tested directly for the clinical delivery of immunotherapies targeting GBM but does hold significant promise. BBB penetrating Nano immunoconjugates have been developed with the aim of crossing the BBB and penetrating intracranial tumors. Galstyan and colleagues (2019) developed anti-CTLA-4 and anti-PD-1 IgG antibodies conjugated to poly (β-L-malic acid), a natural biopolymer scaffold. These Nano immunoconjugates cross the BBB more efficiently than the anti-PD-1 and CTLA-4 IgG antibodies without polymer conjugation, increased the CD4^+^ and CD8^+^ T cell infiltrate into tumors and improved the survival of mice bearing intracranial GL261 tumors compared to those treated with the non-conjugated antibodies (242). Antibody delivery to intracranial tumors can also be improved by disrupting the BBB using focused ultrasound and microbubbles to physically disrupt the tight junctions enabling penetrance of the brain parenchyma. The combination of focused ultrasound (FUS) with microbubbles improves entry of the anti-HER2 antibody Herceptin into brains (243). Similar results have also shown with FUS in combination with microbubbles increases the penetrance of anti-amyloid beta antibodies into the brains of mice in two separate models of Alzheimer’s disease (244). FUS is an attractive option since the opening of BBB is transient (245), thereby minimizing the potential for damage to the brain. More interestingly, FUS itself can be used therapeutically to target intracranial tumors due to its immunomodulation action. Ultrasound waves can expand and contract air bubbles present within cells to generate heat and physically damage cells, inducing cell death, leading to the release of antigenic material and an up-regulation of immune activating molecules such as heat shock proteins (246).

## Concluding Remarks

Glioblastoma multiforme (GBM) is the most frequently occurring primary brain tumor. It is uniformly fatal due to its highly invasive nature and resistance to standard therapies. GBM tumors employ several mechanisms to avoid being detected and killed by immune cells. These include the downregulation of important immune activating molecules such as MHC molecules, as well as upregulating expression of molecules that induce the death of immune cells such as Fas ligand, non-classical MHC molecules such as HLA-E and -G, and PD-L1. GBM cells also secrete numerous immunoinhibitory cytokines such as IL-10, TGF-β, Gal-1, IL-6 and PGE2, to name a few. These cytokines result in the inactivation/death of immune cells as well as the recruitment of inhibitory cells such as regulatory T cells and MDSCs to the TME. These cytokines also lead to a conversion of tumor resident macrophages from the immune activating M1 phenotype to the immunosuppressive M2 phenotype further dampening the anti-tumor immune response.

The plethora of immunosuppressive mechanisms that GBM tumors utilize, as well as their physiological location, make treating them with immunotherapy a daunting task. Although these tumors are immunosuppressive, this immunosuppression can be leveraged to try and boost the anti-tumor immune response. The concept of combining immune checkpoint blockade with active vaccination is one such method that can be used, or the use of genetically modified oncolytic viruses and CAR T cells that actively attack tumors whilst overcoming the local immunosuppression, either via the secretion of immune activating cytokines or immune blocking scFvs. Combinatorial immunotherapy along with improvement of BBB penetration represents an encouraging avenue for GBM therapy in the future. The only caveat to these combined therapies is the possibility of an overactive immune response and potential autoimmunity, this will have to be monitored when moving combinatorial immunotherapy forward.

## Author Contributions

JP wrote the initial draft with SC and SM making significant contributions. All authors contributed to the article and approved the submitted version.

## Funding

This work was supported by the Headcase Cancer Trust (UK), the John and Lucille van Geest Foundation, Nottingham Trent University QR allocation and the John van Geest Cancer Research Centre (Nottingham Trent University).

## Conflict of Interest

Author LD is a director and shareholder of Scancell Ltd and is a named inventor on the ImmunoBody patents.

The remaining authors declare that the research was conducted in the absence of any commercial or financial relationships that could be construed as a potential conflict of interest.
